# A Practical Approach to Assessing the Completeness of Electronic Health Records for Medical Research: Data Quality Study

**DOI:** 10.2196/68935

**Published:** 2026-05-21

**Authors:** Minsik Lim, Doyeon An, Nayeong Son, Woongsang Sunwoo, Suehyun Lee

**Affiliations:** 1 Department of IT Convergence Engineering, Graduate School, Gachon University, Seongnam-si, Gyeonggi‑do, South Korea; 2 Office of Pharmacoepidemiology and big data, Korea Institute of Drug Safety and Risk Management, Anyang, South Korea; 3 Health IT Research Center, Gil Medical Center, Gachon University College of Medicine, Incheon, South Korea; 4 Department of Computer Engineering, Gachon University, 1342 Seongnamdaero, Sujeong‑gu, Seongnam-si, Gyeonggi‑do, 13120, South Korea, 82 031-750-5333

**Keywords:** data quality, quality assessment, completeness, diversity, electronic health record, artificial intelligence, AI

## Abstract

**Background:**

Data quality is the degree to which data are fit for their intended purpose and is described using quality dimensions. The increased use of medical data in clinical research and medical artificial intelligence development has rendered data quality assessment essential. Despite existing data quality definitions, frameworks, and tools, data quality assessment in real-world settings faces multiple challenges. This stems from a lack of understanding of how to assess real-world data quality and interpret the results. Therefore, practical approaches to data quality assessment are needed that are appropriate for diverse data environments, intended uses, quality dimensions, and requirements.

**Objective:**

This study proposes a practical approach for assessing the completeness of electronic health records (EHRs) for medical research. This approach integrates structural completeness, rule-based assessment, and descriptive analyses of completeness and data diversity to clarify how data quality can be measured and meaningfully interpreted in practice.

**Methods:**

The completeness of a large-scale EHR dataset from Gachon University Gil Medical Center was evaluated covering January 2005 to December 2023. Completeness was assessed using a three-part approach comprising (1) structural completeness assessment, (2) rule-based assessment, and (3) descriptive analyses of completeness and data diversity. Assessments were conducted using clinical data quality assessment tools. This practical approach was used to assess EHR completeness for medical research from 1,798,153 patient records.

**Results:**

In the structural assessment, 12.8% (5/39) of the data tables were unavailable, indicating limited capturing of clinician free-text data. The rule-based assessment identified substantial missingness in vocabulary fields (38/124, 30.6%) and missing or special characteristic values in relation to observations (3,643,581/15,313,287, 23.8%), measurements (25,583,622/642,623,715, 4%), care sites (28/1715, 1.6%), and deaths (117/34,330, 0.3%). Descriptive analyses demonstrated a balanced gender distribution (886,489/1,798,153, 49.3% male and 911,664/1,798,153, 50.7% female) and a predominantly Korean racial distribution (1,739,628/1,798,153, 96.7%). Collectively, these findings illustrate the completeness quality of a multiperspective completeness assessment for medical research.

**Conclusions:**

This study demonstrates how data quality dimensions can be measured in practice through a real-world completeness assessment. This practical approach enables evaluation of EHR completeness and provides insights into data quality. Its findings have implications for researchers conducting data quality assessments and applying quality dimensions in medical research.

## Introduction

In the era of big data and artificial intelligence (AI), data are widely recognized as a valuable asset that enables knowledge discovery and value creation [1]. Researchers are increasingly leveraging data-driven insights, the reliability of which depends on data quality, to support decision-making [2,3]. Therefore, high data quality is essential for generating valuable and trustworthy evidence.

Data quality is defined as the degree to which data meet the requirements of their intended use and purpose [4]. It is commonly assessed in terms of quality dimensions, which reflect data characteristics [5,6]. Data quality dimensions describe and quantify how well the data satisfy their set purpose [7].

For example, medical data include information generated throughout the process of medical services, such as diagnoses, examinations, treatments, and surgical procedures [8]. These data are used for health care claims and clinical decision-making to develop patients’ diagnostic and treatment plans [9]. When such uses do not align with the original purpose of data collection, medical data are increasingly reused for broader purposes, including for medical research and AI development [10,11]. Thus, data quality assessment, which evaluates data structures and conditions, is a foundational step for determining whether data are appropriate for their intended use [12].

Various data quality dimensions and assessment frameworks have been proposed to support systematic quality evaluation [5]. In 2016, Kahn et al [13] proposed the harmonized data quality assessment framework to support the standardized use of medical data, which established a shared understanding of the data quality of electronic health records (EHRs) by introducing dimensions and terminology, including completeness (missingness and absence of data), conformance (whether data adhere to predefined formats), and plausibility (credibility or truthfulness of data values).

In 2017, Weiskopf et al [14] proposed a dynamic 3 × 3 framework that operationalizes quality dimensions (complete, correct, and current) across patient, variable, and time perspectives, highlighting the context-dependent nature of data quality and its variability depending on what is measured and when it is measured. Recently, Schwabe et al [15] introduced METRIC, a framework designed for medical AI development and model training, proposing quality characteristics such as measurement process, timeliness, consistency, informativeness, and representativeness.

Additionally, the International Organization for Standardization and International Electrotechnical Commission defined principles for managing data quality across the data life cycle [16,17]. Specifically, the ISO 8000 standard outlines the approach for measuring and managing data quality, and the ISO/IEC 5259 standard provides a data quality framework intended to support data analytics and machine learning [18-20]. Other proposed approaches such as ontology-based definitions of quality dimensions reflect the breadth and diversity of data quality frameworks in the field [21-23].

Despite the definition of quality dimensions and frameworks, standardized and practically implementable methods for evaluating data quality remain limited [24-26]. This indicates an insufficient understanding of how to assess data quality through dimensions and a lack of practical guidance on how to understand data quality in real-world datasets [27,28].

To fill this gap in the literature, this study focused on “completeness,” a fundamental quality dimension that reflects whether required data are fully present and sufficiently captured for a use purpose without bias or loss. Additionally, we evaluated EHR data completeness in a real-world health care setting and derived results using a practical, multi-perspective approach. This study aimed to deepen understanding of how to measure and assess data quality dimensions in practice and provides evidence and guidance that can inform quality management strategies aligned with specific data environments and research objectives.

## Methods

### Overview

**Figure 1.**
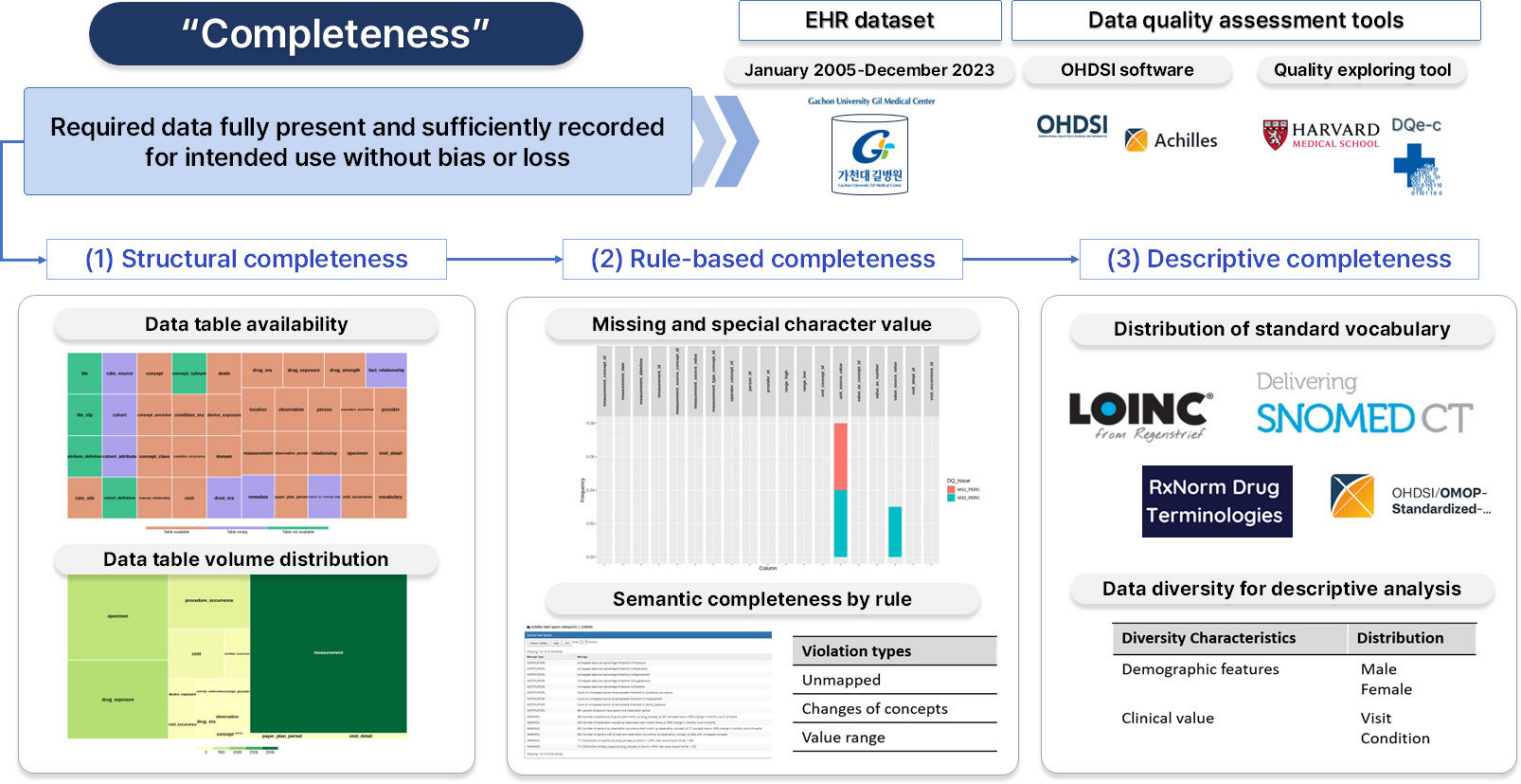
Overview of the completeness assessment workflow applied to the Gachon University Gil Medical Center electronic health record (EHR) dataset from January 2005 to December 2023. Completeness was evaluated using clinical data quality assessment tools. The workflow comprises three complementary components: (1) structural completeness assessment based on table availability and volume, (2) rule-based completeness assessment using SQL validation rules, and (3) descriptive analyses of data distributions to assess both completeness and data diversity. OHDSI: Observational Health Data Sciences and Informatics.

This study used a large-scale EHR from a tertiary university hospital to evaluate data completeness and demonstrate its practical measurement using data quality assessment tools [29]. Figure 1 summarizes the completeness assessment workflow, the study dataset, and the tools used.

### Practical Completeness Assessment Approach

For the completeness assessment, this study applied a practical three-component approach integrating (1) structural assessment, (2) rule-based assessment, and (3) descriptive analyses of completeness and data diversity. First, it assessed structural completeness by verifying database availability and table characteristics, including whether each table was populated, the table status, row counts, and volume. This component provides an overview of database structure and condition before examining missing values and other data issues [30].

Second, the study conducted a rule-based completeness assessment by predefining quality rules according to the data context and characteristics. The rules were implemented as SQL queries executed directly against the database to identify records with missing, invalid, or incomplete values [31]. Table S1 in Multimedia Appendix 1 provides detailed descriptions of the quality rules.

Third, the study analyzed descriptive distributions to assess data diversity and completeness from a researcher perspective. Specifically, it examined baseline characteristics of key variables to understand the coverage of data classes and categories and identify potential imbalances in the study population and recorded events [32,33]. These component results support the interpretation of completeness assessment findings on intended medical research use.

Overall, this multiple practical assessment approach provides actionable guidance for measuring and assessing data completeness in real-world EHR databases.

### Data Quality Assessment Tools

To assess completeness and implement its practical measurement, the study applied established data quality assessment tools to the EHR dataset [34]. It used the Observational Health Data Sciences and Informatics data quality software (ACHILLES) to generate automated database characterization and descriptive statistics for tables and data values [35]. The DQe-c tool was used to examine structural completeness for data availability and distribution for completeness pattern [36-38]. We selected these tools to complement the assessment by enabling evaluation of completeness. Table S2 in Multimedia Appendix 1 provides details regarding tool versions, system specifications, and execution environments.

### EHR Dataset

This study used an EHR-derived dataset from Gachon University Gil Medical Center. The dataset spanned January 2005 to December 2023 and included records from 1,798,153 patients. Table S3 in Multimedia Appendix 1 provides detailed descriptions of the included data tables. This large-scale, real-world dataset provides a robust basis for evaluating completeness and related data quality characteristics in an applied medical research setting.

### Ethical Considerations

This study used secondary, deidentified data from the Gachon University Gil Medical Center EHR, and thus, informed consent was waived. We also confirmed that all data were anonymized and that no individual could be identified. The study protocol was approved by the institutional review board of Gachon University Gil Medical Center (GFIRB2024-243).

## Results

We assessed completeness in the study dataset comprising 1,798,153 patients and approximately 1.67 billion rows of data. Following the practical assessment approach described in the Methods section, results are presented across three components: (1) structural completeness assessment, (2) rule-based completeness assessment, and (3) profiling of completeness and diversity for medical research use.

### Structural Completeness Assessment

**Figure 2.**
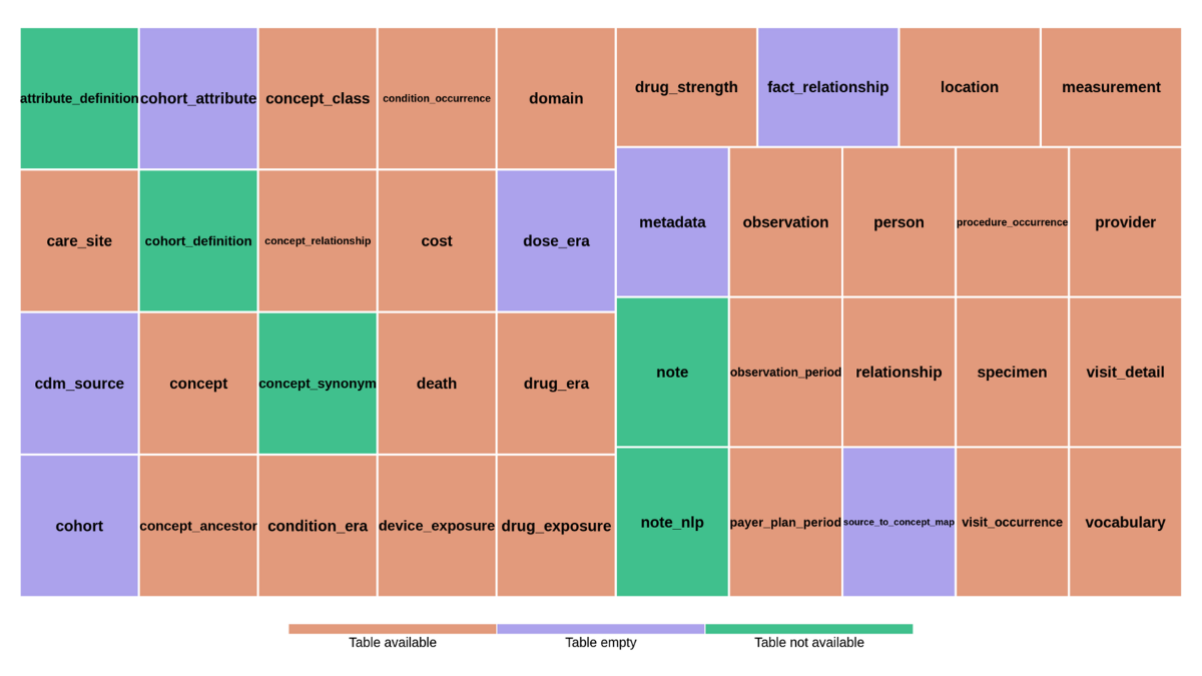
Table availability results from the structural completeness assessment of the database. Table availability was categorized as “available” (orange), “empty” (purple), or “not available” (green). “Not available” indicates that the table was not present in the database and could not be queried. “Empty” indicates that the table existed but contained no records. In this database, 7 tables were empty, and 5 tables were not available, reflecting structural completeness characteristics of the electronic health record database.

This section presents the structural availability and overall scale of the study dataset. As illustrated in Figure 2, the dataset contained 39 tables. Of these 39 tables, 7 (17.9%) were empty, and 5 (12.8%; “note,” “note_nlp,” “attribute_definition,” “cohort_definition,” and “concept_synonym”) were not available. The unavailable “note” and “note_nlp” tables are designed to store unstructured clinical narratives documented by clinicians. The “cohort_definition” and “attribute_definition” tables support the creation and management of study-specific cohorts and their attributes, such as covariates defined through cohort construction algorithms. The “concept_synonym” table stores alternative names and descriptions for concepts. These results indicate the limited availability of free-text documentation and researcher-defined cohort management tables and the unavailability of concept synonym information. Additionally, empty tables such as “metadata” and “dose_era” suggest that certain metadata and derived medication era information were not recorded in the current database. The “metadata” table stores metadata information, and the “dose_era” table represents time spans during which a person is assumed to be exposed to a constant dose of a specific ingredient. The “dose_era” table is typically derived during extract, transform, and load (ETL) from the “drug_exposure” table together with dose information from the “drug_strength” table using standard derivation scripts.

In the structural completeness assessment, the “note” and “note_nlp” tables were unavailable because note data were not collected, and the tables were not implemented in the current database. The “metadata” table was present but contained no records, indicating that dataset metadata were not populated. In particular, the “dose_era” table is a derived table generated during ETL. Therefore, an empty “dose_era” table most likely indicates that the derivation scripts were not executed rather than missing source medication exposure data.

**Figure 3.**
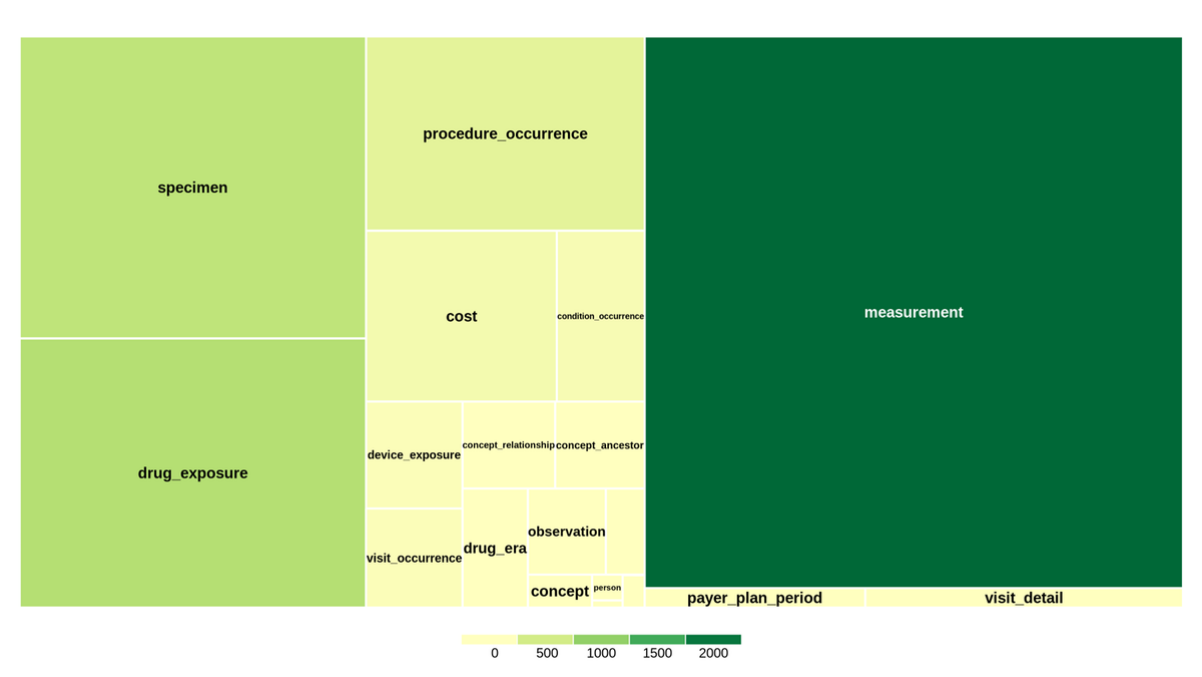
Data table volume (in GB) distribution across available tables in the database. Each rectangle represents a data table; rectangle area is proportional to the table size, and the color indicates the size volume (in GB). This visualization highlights where data volume was concentrated across tables, supporting structural completeness assessment and scoping of research-relevant data domains.

Figure 3 profiles the scale of the available tables in the study database by summarizing data table volume. Among the tables, “measurement”—containing laboratory results, pathological findings, and vital sign measurements—accounted for the largest share of the table volume. The dataset comprised 1,677,839,014 records in total, and a detailed table record count is provided in Table S4 in Multimedia Appendix 1. Notably, large volumes were also observed in the “drug_exposure” table, which captures medication exposure information, and the “specimen” table, which records biological sample information.

These distribution patterns indicate that the dataset was particularly rich in measurement-related data, medication exposure records, and specimen information. This scale profiling provides context for planning use by summarizing table coverage and volume.

### Rule-Based Completeness Assessment

The rule-based completeness assessment applied predefined validation rules to the study database. Table 1 reports results focusing on value presence and rule-defined invalid entities in the data. Textbox 1 presents outputs for extended completeness, which evaluates whether recorded values are semantically sufficient.

**Table 1.** Rule-based completeness assessment results for missing and special character values. The table presents the number and proportion of records flagged based on predefined quality rules for each table-column pair.

Table name	Column name	Records flagged, n/N (%)^a^
Concept_relationship	invalid_reason^b^	55,082,424/55,082,424 (100)
Drug_strength	invalid_reason	2,932,914/2,935,461 (99.9)
Vocabulary	vocabulary_version	38/124 (30.6)
Observation	value_as_string	3,643,581/15,313,287 (23.8)
Measurement	unit_source_value	25,583,622/642,623,715 (4)
Measurement	value_source_value	1,910,233/642,623 (0.3)
Care_site	place_of_service_source_value	28/1715 (1.6)
Care_site	care_site_name	26/1715 (1.5)
Location	state	2/452 (0.4)
Death	cause_source_value	117/34,330 (0.3)
Drug_exposure	dose_unit_source_value	232/166,706,078 (0)

a Records in each table-column pair that were flagged based on the predefined rules as having missing (NULL) values or special character entries.

b The “invalid_reason” field indicates why a relationship or concept was invalidated and can take values of “D” (deleted), “U” (updated), or “NULL” (default). These high-count values represent valid data rather than incompleteness as this field is “NULL” by default.

Textbox 1. **Results of additional rule-based completeness checks. The textbox lists rule outputs related to unmapped concepts, discontinuities in event counts over time, and value range violations in prescription-related rules.**

**Data with unmapped concepts**

- Number of persons by race; data with unmapped concepts
- Number of persons by ethnicity; data with unmapped concepts
- Number of persons in the “death” table by cause_concept_id; data with unmapped concepts
- Number of persons with at least one drug exposure by drug_concept_id; data with unmapped concepts
- Number of persons with at least one procedure occurrence by procedure_concept_id; data with unmapped concepts
- Number of persons with at least one condition occurrence by condition_concept_id; data with unmapped concepts
- Number of persons with at least one condition era by condition_concept_id; data with unmapped concepts
- Number of persons with at least one observation occurrence by observation_concept_id; data with unmapped concepts

**Changes in concepts**

- Number of persons by drug exposure start month by drug_concept_id; 1059 concepts had a 100% change in monthly count of events
- Number of persons by drug era start month by drug_concept_id; 567 concepts had a 100% change in monthly count of events
- Number of persons by procedure occurrence start month by procedure_concept_id; 401 concepts had a 100% change in monthly count of events
- Number of persons by condition occurrence start month by condition_concept_id; 703 concepts had a 100% change in monthly count of events
- Number of persons by condition era start month by condition_concept_id; 875 concepts had a 100% change in monthly count of events
- Number of persons by observation occurrence start month by observation_concept_id; 27 concepts had a 100% change in monthly count of events

**Value range**

- Distribution of days_supply by drug_concept_id; maximum value should not be >180
- Distribution of quantity by drug_concept_id; maximum value should not be >600

As shown in Table 1, notable findings required interpretation based on dataset semantics. In the “concept_relationship” and “drug_strength” tables, the “invalid_reason” column is “NULL” by default and is populated only when a relationship or mapping has been invalidated. Therefore, the predominance of “NULL” values in this column data does not indicate incompleteness.

However, several columns showed missingness or special character values. In the “vocabulary” table, “vocabulary_version,” which stores released vocabulary version information, was missing in 30.6% of records (38/124). In the “observation” table, “value_as_string” column, which stores observation values recorded as text, showed substantial missing data, with 23.8% (3,643,581/15,313,287) of records flagged as incomplete.

In the “measurement” table, “unit_source_value” and “value_source_value,” which store measurement units and source result values, respectively, contained special character entries at relatively low rates, indicating that several source units or values were carried forward without sufficient normalization. Special characters were also detected at very low rates in several source value fields, including “place_of_service_source_value,” “care_site_name,” “state,” “cause_source_value,” and “dose_unit_source_value.” These results indicate localized nonstandard formatting in several source value fields, including location, death, and drug exposure tables.

Textbox 1 highlights 3 types of rule findings. First, the study identified unmapped concept patterns in multiple domains, including demographic fields such as gender, race, and ethnicity, and event-based tables. These results indicate that values in the source data are not fully represented using standard concepts, limiting their interpretability and consistent use for research. Second, the rules flagged abrupt shifts in event count for condition occurrence, procedure occurrence, and drug exposure. These abrupt changes indicate breaks in the temporal continuity of data recording or loading. From a completeness perspective, events may have been incompletely captured during specific periods. Third, value range checks identified data such as “days_supply” and “quantity” exceeding predefined bounds. These out-of-range values suggest that a subset of records may not align with expected clinical patterns or may reflect data transformation issues, which can reduce the reliability of downstream analyses that depend on these fields.

These rule-based findings demonstrate that completeness assessment can address not only whether values are present but also whether data meet use conditions and semantic meaning, facilitating the detection and identification of major quality issues.

### Completeness and Diversity for Medical Research

As part of descriptive profiling to support the research purpose, this study examined the distribution of mapped standard vocabularies across key clinical domains. EHR data are commonly standardized using terminologies such as Logical Observation Identifiers Names and Codes and Systematized Nomenclature of Medicine–Clinical Terms (SNOMED CT); however, mapping coverage can vary according to clinical and institutional practice [39]. Understanding the mapped vocabulary landscape is important for planning data use and interpreting the data standard [40].

Table 2 summarizes the distribution of standard vocabularies used by data across clinical domains. In the “measurement” table, most records were mapped to Logical Observation Identifiers Names and Codes (468,274,853/642,638,656, 72.9%), followed by SNOMED CT (116,316,640/642,638,656, 18.1%). In the “drug_exposure” table, mappings were dominated by RxNorm Extension (143,512,501/166,702,480, 86.1%) with additional use of RxNorm (16,810,245/166,702,480, 10.1%). In the “observation” table, the mapped concepts were predominantly SNOMED CT (14,833,557/15,313,463, 96.9%). In the “procedure_occurrence” table, mappings were distributed across Observational Medical Outcomes Partnership standardized vocabularies (85,344,562/159,014,912, 54.0%) and SNOMED CT (69,604,840/159,014,912, 44.1%).

**Table 2.** Distribution of mapped standard vocabularies across major clinical domains. Counts and proportions are reported for the primary vocabularies used to represent concepts in the “measurement,” “drug_exposure,” “observation,” and “procedure_occurrence” tables in the study dataset.

Table name and vocabulary	Rows, n (%)	
Measurement (n=642,623,715)		
LOINC^a^	468,274,853 (72.9)	
SNOMED CT^b^	116,316,640 (18.1)	
OMOP^c^ standardized vocabularies	52,320,453 (8.1)	
Other	5,726,710 (0.9)	
Drug_exposure (n=166,706,078)		
RxNorm Extension	143,512,501 (86.1)	
RxNorm	16,810,245 (10.1)	
OMOP standardized vocabularies	4,026,859 (2.4)	
Other	2,352,875 (1.4)	
Observation (n=15,313,287)		
SNOMED CT	14,833,557 (96.9)	
LOINC	399,874 (2.6)	
Other	80,032 (0.5)	
Procedure_occurrence (n=159,007,634)		
OMOP standardized vocabularies	85,344,562 (53.7)	
SNOMED CT	69,604,840 (43.8)	
Other	4,065,510 (2.6)	

a LOINC: Logical Observation Identifiers Names and Codes.

b SNOMED CT: Systematized Nomenclature of Medicine–Clinical Terms.

c OMOP: Observational Medical Outcomes Partnership.

Examining these vocabulary distributions provides a foundational understanding of the dataset structure for research use.

Descriptive diversity profiling characterized the study cohort by describing how records were distributed across demographic groups and clinical contexts, and it was used to summarize population characteristics and recording patterns. There is no gold-standard distribution for these profiles because the expected distribution depends on the intended use of the data. However, when a study requires specific subpopulations or clinical settings, evaluating whether the dataset provides sufficient representation of these target populations is an essential component of data quality assessment for medical research. Thus, limited diversity may indicate that the dataset does not meet the requirements for its intended use.

Table 3 presents descriptive diversity profiling results for the study dataset with gender, race, ethnicity, and clinical characteristics. The gender distribution was balanced, with male patients accounting for 49.3% (886,489/1,798,153) and female patients accounting for 50.7% (911,664/1,798,153) of the dataset. In contrast, race and ethnicity information showed substantial skewness and limited standardization. Race was predominantly mapped to Korean, which is expected given the national context of the data source. However, a small proportion of records were labeled as “No matching concept,” suggesting either the presence of non-Korean race values or incomplete mapping to standard concepts. Ethnicity was not mapped to a standard concept for the entire dataset and was recorded as “No matching concept.” This is because the ethnicity field is primarily intended to distinguish “Hispanic or Latino” from “Not Hispanic or Latino.” This field was designed for US-based datasets to support ethnicity classification and is typically not applicable or routinely collected in Asian settings, including South Korea. Therefore, the observed ethnicity results should be interpreted not as a data collection quality issue but as a reflection of the US-centric design and intended use of the data model.

**Table 3.** Descriptive profiling of data diversity for research use. The table summarizes the distribution of key demographic attributes in the study patients and the most frequently recorded categories for visit type, condition, drug exposure, and measurement concept by person (n=1,798,153).

Diversity characteristic	Patients, n (%)
Sex	
Male	886,489 (49.3)
Female	911,664 (50.7)
Race	
Korean	1,739,628 (96.7)
No matching concept	58,525 (3.3)
Ethnicity	
No matching concept	1,798,153 (100)
Visit type	
Outpatient visit	1,444,995 (80.4)
Emergency room visit	687,254 (38.2)
Inpatient visit	494,492 (27.5)
Health examination	272,061 (15.1)
Intensive care	46,651 (2.6)
Condition type	
Inflammatory disorder of the digestive tract	134,783 (7.5)
Essential hypertension	115,262 (6.4)
Gastritis	111,126 (6.2)
Drug exposure type	
100-mL sodium chloride 9–mg/mL injectable solution (JW NS)	360,530 (20.1)
10,000-mL oxygen gas for inhalation	358,831 (20.0)
*Artemisia argyi* leaf extract 60-mg oral tablet (Stillen)	282,130 (15.7)
Measurement type	
Hemoglobin (mass per volume) in blood by calculation	1,068,822 (59.4)
Alanine aminotransferase (enzymatic activity per volume) in serum or plasma	1,037,714 (57.7)
Aspartate aminotransferase (enzymatic activity per volume) in serum or plasma	1,037,534 (57.7)
Creatinine (mass per volume) in serum or plasma	1,005,168 (55.9)

Additionally, through determining the most frequently recorded categories across the dataset, it was possible to characterize where data capture was concentrated. Most visit-related records were generated from outpatient encounters, whereas records associated with intensive care were comparatively rare. This distribution indicates that the dataset primarily reflects limited representation of intensive care populations, which is important to consider when defining research aims and target studies [41]. For the condition, drug exposure, and measurement domains, the most frequently recorded categories were identified to provide practical context for understanding baseline data coverage and typical clinical content in the database.

These distribution summaries support the interpretation of data class completeness and understanding of diversity by documenting demographic information and identifying domains where data are concentrated.

## Discussion

### Principal Findings

A key finding from the structural completeness assessment was that the database did not contain “note” and “note_nlp” tables, indicating that clinical free-text narratives were not available. Because clinical notes are increasingly used for studies based on natural language processing (NLP) and large language models (LLMs), this limits the dataset’s suitability for NLP and LLM study purposes [42]. Although some text information may exist in other tables, NLP research requires clinical narrative text [43]. Therefore, establishing a systematic framework for collecting and constructing incomplete data is necessary.

In the rule-based assessment, we applied detailed validation rules to examine completeness across multiple tables and columns. However, we also found that rule outputs cannot be interpreted without understanding the meaning of each field and the data context. For example, “invalid_reason” appeared to be missing in most records, but “NULL” was the default value. This highlights that completeness assessment is not only about running rules but also about interpreting results based on data characteristics and semantics. Additionally, the detection of unmapped concepts and abrupt changes in monthly events indicate crucial data quality issues. These findings illustrate that rule-based assessment must extend beyond value presence to evaluate the integrity of the data quality. Specifically, by identifying unmapped patterns, temporal shifts, and out-of-range values, we can pinpoint systematic data quality issues such as capture failures or transformation errors. This assessment approach serves as a diagnostic warning system to identify specific types of issues and helps examine data quality from missing values to semantic issues [31,44]. However, interpreting the outputs requires an understanding of data. As illustrated by the “invalid_reason” example in our results, rule outputs do not always reflect the same meaning. Therefore, rule-based assessment requires expertise to implement rules and interpret the outcomes. It also requires review of the rules to ensure validity and meaningfulness in the assessment [45].

Medical research should conduct descriptive diversity profiling to characterize the study cohort by quantifying how records are distributed across demographic groups and clinical contexts [32]. This approach does not rely on a single ideal distribution because a dataset can still be complete even when certain encounter types are absent if the data source is designed not to capture them. However, when the intended use requires specific subpopulations or care settings, limited representation can constrain research feasibility and may contribute to biased findings in medical research and model behavior in medical AI [46]. Therefore, diversity should be interpreted not only as a description of data distribution but also as an indicator of whether the dataset sufficiently covers the target populations and clinical contexts needed for the intended use. In other words, these data quality assessment results underscore that data quality should be assessed and interpreted according to the definition of data quality, dimensions, and criteria; measurement methods; and intended use of the dataset.

The principal findings have broader implications for medical informatics by illustrating how completeness challenges emerge in real-world EHR data. To contextualize our approach, we compared our completeness assessment with prior data quality studies in Asia and with international Observational Medical Outcomes Partnership–related work [47-49]. Previous studies have largely relied on Observational Health Data Sciences and Informatics tools to characterize datasets and flag rule-based issues. In contrast, this study focused on completeness for medical research and operationalized it using three complementary components: (1) structural completeness assessment, (2) rule-based validation, and (3) descriptive profiling. We further interpreted the assessment outputs in relation to data generation processes, ETL decisions, and analytic intent rather than treating the results as simple quality status indicators. This comparison suggests that completeness assessment for secondary use should extend beyond issue detection to include interpretation and cause-oriented reasoning because these issues directly affect data reusability and comparability.

Specifically, the unavailability of clinical note data in our dataset can be interpreted as an infrastructure and representation gap for clinical narratives rather than as a simple missing data issue [50]. This finding reflects real-world barriers to collecting and standardizing unstructured clinical text and transforming it into structured representations for secondary use. These barriers have motivated ongoing work on clinical text processing and NLP and LLM integration approaches for clinical note generation [51]. As the practical feasibility of these approaches increases, evaluating the reliability and usability of AI-generated narratives becomes an additional data quality requirement [52]. Additionally, mapping gaps and incomplete standardization issues highlight the need for continued ETL process improvement and tool development to support reliable transformation in real-world EHR databases [53,54].

Collectively, these findings indicate that data quality assessment is not an end point. It is part of an iterative process in which quality findings lead to remediation efforts such as refining ETL and mapping workflows or generating synthetic data. The outputs of these remediation steps should then be re-evaluated for reliability, usability, and fitness for the intended secondary use.

Together, these findings suggest that this case study can offer broad data quality insights for improving EHR reusability.

### Data Quality

Data quality is a foundational prerequisite, and numerous frameworks have been proposed to define quality dimensions, terminology, and evaluation perspectives.

However, translating these concepts into practical assessment and interpretation in real-world settings remains challenging. Although many frameworks provide a shared language for describing data quality, it is difficult to connect framework concepts to practical data quality assessments [55]. For example, medical AI frameworks emphasize quality characteristics relevant to model development, yet these considerations are not incorporated into dataset curation and training pipelines [15].

Ultimately, achieving meaningful data quality requires a holistic approach that integrates quality definitions, dimensions, and frameworks with practical assessment methods and tools. Appropriate interpretation of results, together with continuous efforts and monitoring, is necessary for quality management.

### Strengths and Limitations

A key strength of this study is its completeness evaluation of a large-scale EHR dataset in which completeness was assessed using multiple complementary perspectives and the findings were interpreted in context. By providing an empirical example of how completeness can be measured in practice, this study offers a practical reference for researchers planning completeness assessments and supports efforts to improve data quality.

This study also has limitations. Because data quality is context dependent and may be defined and operationalized differently depending on purpose and perspective, our assessment focused primarily on completeness and did not evaluate other quality dimensions or frameworks. Completeness cannot be fully captured by a single set of rules or metrics across all research contexts. Thus, the specificity and depth of data quality findings can vary substantially by assessment method and tool. This reflects the wide variation in underlying frameworks, dimensions, criteria, and tool capabilities in real-world data quality evaluation, making assessment outputs inherently heterogeneous.

### Conclusions

This study operationalized and assessed data completeness in a large-scale EHR dataset. A practical approach was applied consisting of structural completeness assessment, rule-based validation, and descriptive statistical profiling, and completeness findings were derived from each component. This completeness assessment workflow demonstrates how data quality can be measured and interpreted in real-world clinical settings.

In conclusion, this study emphasizes that data quality work requires not only quality definitions but also practical assessment methods and interpretable outputs. By presenting a methodology-based assessment workflow and results from a real clinical dataset, it provides a concrete reference for applying data quality dimensions in medical research settings. These results and insights provide a representative case for data quality research.

## Supplementary material

10.2196/68935 Multimedia Appendix 1 Detailed quality rules for data completeness assessment; detailed data quality assessment tool version, system specification, and environment; specific description of the data tables for the study; and detailed results of data row count for structural completeness assessment (n=1,677,839,014).

## Abbreviations

AI: artificial intelligence
CT: Systematized Nomenclature of Medicine–Clinical Terms
EHR: electronic health record
ETL: extract, transform, and load
LLM: large language model
NLP: natural language processing

## References

[1] Paul S, Riffat M, Yasir A (2021). Industry 4.0 applications for medical/healthcare services. J Sens Actuator Netw.

[2] Pastorino R, De Vito C, Migliara G (2019). Benefits and challenges of big data in healthcare: an overview of the European initiatives. Eur J Public Health.

[3] Fraser HSF, Mugisha M, Bacher I (2024). Factors influencing data quality in electronic health record systems in 50 health facilities in Rwanda and the role of clinical alerts: cross-sectional observational study. JMIR Public Health Surveill.

[4] Wang RY, Strong DM (1996). Beyond accuracy: what data quality means to data consumers. J Manag Inf Syst.

[5] Lewis AE, Weiskopf N, Abrams ZB (2023). Electronic health record data quality assessment and tools: a systematic review. J Am Med Inform Assoc.

[6] An D, Lim M, Lee S (2025). Challenges for data quality in the clinical data life cycle: systematic review. J Med Internet Res.

[7] Bönisch C, Schmidt C, Kesztyüs D, Kestler HA, Kesztyüs T (2025). Proposal for using AI to assess clinical data integrity and generate metadata: algorithm development and validation. JMIR Med Inform.

[8] Dawson DE (2006). National Emergency Medical Services Information System (NEMSIS). Prehosp Emerg Care.

[9] Mayo CS, Yao J, Eisbruch A (2017). Incorporating big data into treatment plan evaluation: development of statistical DVH metrics and visualization dashboards. Adv Radiat Oncol.

[10] Köpcke F, Trinczek B, Majeed RW (2013). Evaluation of data completeness in the electronic health record for the purpose of patient recruitment into clinical trials: a retrospective analysis of element presence. BMC Med Inform Decis Mak.

[11] Rajpurkar P, Chen E, Banerjee O, Topol EJ (2022). AI in health and medicine. Nat Med.

[12] Pezoulas VC, Kourou KD, Kalatzis F (2019). Medical data quality assessment: on the development of an automated framework for medical data curation. Comput Biol Med.

[13] Kahn MG, Callahan TJ, Barnard J (2016). A harmonized data quality assessment terminology and framework for the secondary use of electronic health record data. EGEMS (Wash DC).

[14] Weiskopf NG, Bakken S, Hripcsak G, Weng C (2017). A data quality assessment guideline for electronic health record data reuse. EGEMS (Wash DC).

[15] Schwabe D, Becker K, Seyferth M, Klaß A, Schaeffter T (2024). The METRIC-framework for assessing data quality for trustworthy AI in medicine: a systematic review. NPJ Digit Med.

[16] Oviedo J, Rodriguez M, Trenta A, Cannas D, Natale D, Piattini M (2024). ISO/IEC quality standards for AI engineering. Comput Sci Rev.

[17] (2017). 24765-2017 - ISO/IEC/IEEE International Standard - Systems and Software Engineering--Vocabulary.

[18] Rivas BR, Merino J, Caballero I, Serrano MA, Piattini M (2016). Towards a service architecture for master data exchange based on ISO 8000 with support to process large datasets. Comput Stand Interfaces.

[19] Aiyankovil KG, Lewis D, Hernandez J Mapping data governance requirements between the European Union’s AI Act and ISO/IEC 5259: a semantic analysis. https://openreview.net/forum?id=5ilvDyTkbg&filter=excludedInvitations%3ASEMANTiCS.cc%2F2024%2FW.

[20] Knyazev AV, Cheremukhina JJ (2024). 2024 International Conference “Quality Management, Transport and Information Security, Information Technologies.”.

[21] Liaw ST, Rahimi A, Ray P (2013). Towards an ontology for data quality in integrated chronic disease management: a realist review of the literature. Int J Med Inform.

[22] Yu Y, Khadivi S, Xu J (2022). Proceedings of the 29th International Conference on Computational Linguistics.

[23] Coburn CE, Turner EO (2011). Research on data use: a framework and analysis. Meas Interdiscip Res Perspect.

[24] Huser V, Li X, Zhang Z (2019). Extending Achilles Heel data quality tool with new rules informed by multi-site data quality comparison. Stud Health Technol Inform.

[25] Huser V, DeFalco FJ, Schuemie M (2016). Multisite evaluation of a data quality tool for patient-level clinical data sets. EGEMS (Wash DC).

[26] Blasco-Calafat A, Blanes-Selva V, Fragner T (2025). Multisource coherence analysis of the first European multicenter cohort study for cancer prevention in people experiencing homelessness: data quality study. JMIR Med Inform.

[27] Bian J, Lyu T, Loiacono A (2020). Assessing the practice of data quality evaluation in a national clinical data research network through a systematic scoping review in the era of real-world data. J Am Med Inform Assoc.

[28] Cho S, Weng C, Kahn MG, Natarajan K (2021). Identifying data quality dimensions for person-generated wearable device data: multi-method study. JMIR Mhealth Uhealth.

[29] Lee S, Choi YH, Kim HM (2025). The Cancer Clinical Library Database (CCLD) from the Korea-Clinical Data Utilization Network for Research Excellence (K-CURE) project. Cancer Res Treat.

[30] Ji H, Kim S, Yi S, Hwang H, Kim JW, Yoo S (2020). Converting clinical document architecture documents to the common data model for incorporating health information exchange data in observational health studies: CDA to CDM. J Biomed Inform.

[31] Wang Z, Talburt JR, Wu N, Dagtas S, Zozus MN (2020). A rule-based data quality assessment system for electronic health record data. Appl Clin Inform.

[32] Zhou X, Li T, Hayama H (2025). Diagnosis of cardiac conditions from 12-lead electrocardiogram through natural language supervision. NPJ Digit Med.

[33] Hofmanninger J, Prayer F, Pan J, Röhrich S, Prosch H, Langs G (2020). Automatic lung segmentation in routine imaging is primarily a data diversity problem, not a methodology problem. Eur Radiol Exp.

[34] Ozonze O, Scott PJ, Hopgood AA (2023). Automating electronic health record data quality assessment. J Med Syst.

[35] Dixon BE, Wen C, French T, Williams JL, Duke JD, Grannis SJ (2020). Extending an open-source tool to measure data quality: case report on Observational Health Data Science and Informatics (OHDSI). BMJ Health Care Inform.

[36] Estiri H, Stephens KA, Klann JG, Murphy SN (2018). Exploring completeness in clinical data research networks with DQe-c. J Am Med Inform Assoc.

[37] Klann JG, Henderson DW, Morris M (2023). A broadly applicable approach to enrich electronic-health-record cohorts by identifying patients with complete data: a multisite evaluation. J Am Med Inform Assoc.

[38] Estiri H, Klann JG, Weiler SR (2019). A federated EHR network data completeness tracking system. J Am Med Inform Assoc.

[39] Dong H, Falis M, Whiteley W (2022). Automated clinical coding: what, why, and where we are?. NPJ Digit Med.

[40] Ryu H, Sung S, Park K (2025). Comparative study of LOINC and SNOMED CT in panel mapping: enhancing interoperability in laboratory testing. Int J Med Inform.

[41] Johnson AE, Pollard TJ, Shen L (2016). MIMIC-III, a freely accessible critical care database. Sci Data.

[42] Han J, Park J, Huh J, Oh U, Do J, Kim D (2024). CHI EA ’24: Extended Abstracts of the CHI Conference on Human Factors in Computing Systems.

[43] Palm E, Manikantan A, Mahal H, Belwadi SS, Pepin ME (2025). Assessing the quality of AI-generated clinical notes: validated evaluation of a large language model ambient scribe. Front Artif Intell.

[44] Blacketer C, Voss EA, DeFalco F (2021). Using the data quality dashboard to improve the EHDEN network. Appl Sci.

[45] Marteau BL, Hornback A, Zhong Y Improving a large healthcare system research data warehouse using OHDSI’s data quality dashboard.

[46] Gong Y, Liu G, Xue Y, Li R, Meng L (2023). A survey on dataset quality in machine learning. Inf Softw Technol.

[47] Yoon D, Ahn EK, Park MY (2016). Conversion and data quality assessment of electronic health record data at a Korean tertiary teaching hospital to a common data model for distributed network research. Healthc Inform Res.

[48] Julian GS, Shau WY, Chou HW, Setia S (2024). Bridging real-world data gaps: connecting dots across 10 Asian countries. JMIR Med Inform.

[49] Blacketer C, Defalco FJ, Ryan PB, Rijnbeek PR (2021). Increasing trust in real-world evidence through evaluation of observational data quality. J Am Med Inform Assoc.

[50] Keloth VK, Banda JM, Gurley M (2023). Representing and utilizing clinical textual data for real world studies: an OHDSI approach. J Biomed Inform.

[51] Yuan D, Rastogi E, Naik G (2024). Proceedings of the 2024 Conference of the North American Chapter of the Association for Computational Linguistics: Human Language Technologies.

[52] Dahlberg A, Käenniemi T, Winther-Jensen T (2026). Measuring the quality of AI-generated clinical notes: a systematic review and experimental benchmark of evaluation methods. Artif Intell Med.

[53] Yu Y, Zong N, Wen A (2022). Developing an ETL tool for converting the PCORnet CDM into the OMOP CDM to facilitate the COVID-19 data integration. J Biomed Inform.

[54] Peng Y, Henke E, Reinecke I, Zoch M, Sedlmayr M, Bathelt F (2023). An ETL-process design for data harmonization to participate in international research with German real-world data based on FHIR and OMOP CDM. Int J Med Inform.

[55] Schmidt CO, Struckmann S, Enzenbach C (2021). Facilitating harmonized data quality assessments. A data quality framework for observational health research data collections with software implementations in R. BMC Med Res Methodol.

