# Supplementary material for: A Practical Approach to Assessing the Completeness of Electronic Health Records for Medical Research: Data Quality Study
Source: JMIR Med Inform. 2026 May 21;14:e68935. doi: 10.2196/68935 (PMC13193671; doi:10.2196/68935)
Supplement: Multimedia Appendix 1 [file medinform-v14-e68935-s001.docx]

Multimedia Appendix

**Multimedia Appendix Caption**

**Table 1.** Detailed quality rules for data completeness assessment.

**Table 2**. Detailed data quality assessment tools version, system specification and environment.

**Table 3.** A specific description of data table for study.

**Table 4.** Detailed results of data rows count for structural completeness assessment (n=1,677,839,014).

**Table 1.** Detailed quality rules for data completeness assessment.

| **Category** | **Rule ID** | **Analysis item** |
| --- | --- | --- |
| Person | 2 | Number of persons by gender |
| Person | 4 | Number of persons by race |
| Person | 5 | Number of persons by ethnicity |
| Person | 9 | Number of persons with invalid care_site_id |
| Condition Occurrence | 400 | Number of persons with at least one condition occurrence, by condition_concept_id |
| Condition Occurrence | 402 | Number of persons by condition occurrence start month, by condition_concept_id |
| Death | 500 | Number of persons with death, by cause_concept_id |
| Procedure Occurrence | 600 | Number of persons with at least one procedure occurrence, by procedure_concept_id |
| Procedure Occurrence | 602 | Number of persons by procedure occurrence start month, by procedure_concept_id |
| Drug Exposure | 700 | Number of persons with at least one drug exposure, by drug_concept_id |
| Drug Exposure | 702 | Number of persons by drug exposure start month, by drug_concept_id |
| Drug Exposure | 715 | Distribution of days_supply by drug_concept_id |
| Drug Exposure | 717 | Distribution of quantity by drug_concept_id |
| Observation | 800 | Number of persons with at least one observation occurrence, by observation_concept_id |
| Observation | 802 | Number of persons by observation occurrence start month, by observation_concept_id |
| Observation | 820 | Number of observation records by observation start month |
| Drug Era | 902 | Number of persons by drug era start month, by drug_concept_id |
| Condition Era | 1,000 | Number of persons with at least one condition era, by condition_concept_id |
| Condition Era | 1,002 | Number of persons by condition era start month, by condition_concept_id |

**Table 2.** Detailed data quality assessment tools version, system specification and environment.

| **Category** | **Item** | **Description** |
| --- | --- | --- |
| Server environment | DBMS type | PostgreSQL |
|  | Database name | Postgres |
|  | CDM version | GILMC_5.3.1_weekly |
|  | Schema name | OMOP_CDM |
| Network setup | Network type | Internal hospital network |
|  | Connection method | Local access |
| Tools and versions | OHDSI | ACHILLES v1.7.2 |
|  | DQe-c | DQe-c-v2 |
| Computing resource | Server CPU | i9-10900 |
|  | Memory (RAM) | DDR4 16G |
|  | Storage (Disk) | 1.5T |
| Performance environment | Analysis environment | Gachon University Gil Hospital Health IT Center |
|  | Performing organization | Gachon University |

**Table 3.** A specific description of data table for study.

| **Table** | **Description** |
| --- | --- |
| Note | Stores unstructured clinical free-text notes. |
| Note_nlp | Stores natural language processing (NLP) outputs derived from unstructured clinical free text. |
| cohort | Stores subjects/patients who meet the specified criteria. |
| Cohort_attribute | Stores associated information (attributes) for cohort members. |
| Cohort_definition | Stores information related to the definition/specification of cohorts. |
| Attribute_definition | Stores covariate and attribute information for cohort members. |
| Concept_synonym | Stores synonyms for concepts and vocabulary codes. |
| Dose_era | Stores information on periods of drug exposure for a patient. |
| Metadata | Stores metadata information for the dataset. |
| Fact_relationship | Stores relationship information between facts recorded across multiple CDM tables (e.g., person-to-person relationships, hierarchical organization of care sites, relationships between diseases/conditions). |
| Source_to_concept_map | Stores mappings between source codes (e.g., HIS source codes) used in the ETL process and standard medical data concepts. |

**Table 4**. Detailed results of data rows count for structural completeness assessment (n=1,677,839,014).

| **Table** | **Rows** |
| --- | --- |
| Measurement | 642,638,656 |
| Specimen | 286,324,576 |
| Drug_exposure | 166,702,480 |
| Procedure_occurrence | 159,014,912 |
| Concept_ancestor | 79,923,200 |
| Cost | 60,832,184 |
| Concept_relationship | 55,082,424 |
| Drug_era | 45,648,264 |
| Condition_occurrence | 38,943,560 |
| Device_exposure | 31,777,746 |
| Condition_era | 23,162,044 |
| Visit_occurrence | 23,082,424 |
| Visit_detail | 23,082,310 |
| Observation | 15,313,463 |
| Payer_plan_period | 11,044,029 |
| Concept | 8,680,800 |
| Drug_strength | 2,935,461 |
| Observation_period | 1,798,153 |
| Person | 1,798,153 |
| Death | 34,330 |
| Provider | 16,435 |
| Care_site | 1,715 |
| Relationship | 656 |
| Location | 452 |
| Concept_class | 415 |
| Vocabulary | 124 |
| Domain | 48 |
